# The effect of concomitant peripheral injury on traumatic brain injury pathobiology and outcome

**DOI:** 10.1186/s12974-016-0555-1

**Published:** 2016-04-26

**Authors:** Stuart J. McDonald, Mujun Sun, Denes V. Agoston, Sandy R. Shultz

**Affiliations:** Department of Physiology, Anatomy and Microbiology, La Trobe University, Melbourne, VIC Australia; Department of Medicine, The Royal Melbourne Hospital, The University of Melbourne, Parkville, VIC Australia; Department of Anatomy, Physiology, and Genetics, Uniformed Services University of the Health Sciences, Bethesda, MD USA

**Keywords:** Multitrauma, Polytrauma, Traumatic brain injury, Inflammation, Concussion, Animal model, Clinical, Cytokines, Bone fracture

## Abstract

**Background:**

Traumatic injuries are physical insults to the body that are prevalent worldwide. Many individuals involved in accidents suffer injuries affecting a number of extremities and organs, otherwise known as multitrauma or polytrauma. Traumatic brain injury is one of the most serious forms of the trauma-induced injuries and is a leading cause of death and long-term disability. Despite over dozens of phase III clinical trials, there are currently no specific treatments known to improve traumatic brain injury outcomes. These failures are in part due to our still poor understanding of the heterogeneous and evolving pathophysiology of traumatic brain injury and how factors such as concomitant extracranial injuries can impact these processes.

**Main body:**

Here, we review the available clinical and pre-clinical studies that have investigated the possible impact of concomitant injuries on traumatic brain injury pathobiology and outcomes. We then list the pathophysiological processes that may interact and affect outcomes and discuss promising areas for future research. Taken together, many of the clinical multitrauma/polytrauma studies discussed in this review suggest that concomitant peripheral injuries may increase the risk of mortality and functional deficits following traumatic brain injury, particularly when severe extracranial injuries are combined with mild to moderate brain injury. In addition, recent animal studies have provided strong evidence that concomitant injuries may increase both peripheral and central inflammatory responses and that structural and functional deficits associated with traumatic brain injury may be exacerbated in multiply injured animals.

**Conclusions:**

The findings of this review suggest that concomitant extracranial injuries are capable of modifying the outcomes and pathobiology of traumatic brain injury, in particular neuroinflammation. Though additional studies are needed to further identify the factors and mechanisms involved in central and peripheral injury interactions following multitrauma and polytrauma, concomitant injuries should be recognized and accounted for in future pre-clinical and clinical traumatic brain injury studies.

## Background

Traumatic injuries caused by physical insults to the body are a common and serious medical problem. Because of the high-impact nature of trauma-inducing accidents, patients commonly suffer concomitant injuries to multiple body regions and organs, otherwise known as multitrauma or polytrauma. Amongst the most devastating of trauma injuries, traumatic brain injury (TBI) is a leading cause of death and morbidity worldwide. To date, there is still no treatment known to improve TBI outcomes, which is in large part due to our poor understanding of its evolving pathophysiology, and how factors such as concomitant peripheral injuries can impact these processes. In light of the ongoing failures in clinical trials in TBI, and the heavy burden of TBI on society, factors like concomitant extracranial injuries must be acknowledged and studied as we strive to improve the outcome of TBI patients. Here, we review the available clinical and pre-clinical studies that have investigated the possible impact of concomitant injuries on TBI pathobiology and outcomes, summarize the potential interactive pathophysiologies with a particular focus on neuroinflammation, and discuss potential areas for future research.

**Fig. 1:**
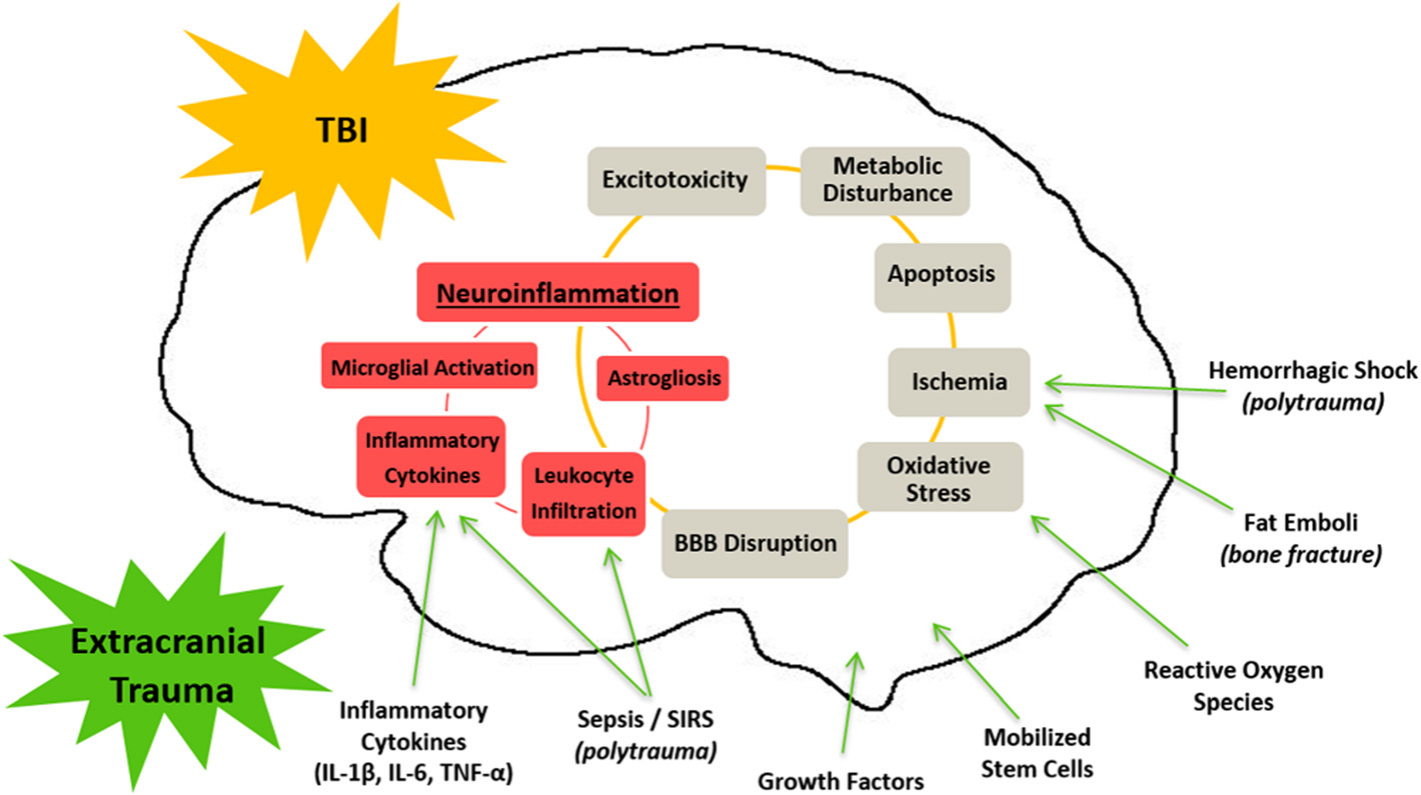
Possible pathways through which extracranial injury may alter TBI pathobiology. Secondary injury processes of TBI include neuroinflammation, excitotoxicity, metabolic disturbances, apoptosis, ischemia, oxidative stress, and BBB disruption. The neuroinflammatory response of TBI is characterized by microglial and astrocyte activation, leukocyte infiltration and elevated levels of pro-inflammatory cytokines. For multitrauma/polytrauma patients, there is potential for the systemic effects of significant extracranial injuries to impact upon secondary injury pathways of TBI, and in particular the neuroinflammatory response. Possible extracranial trauma-induced influences on TBI include elevated circulating inflammatory cytokines, growth factors, reactive oxygen species, and for the patient with bone fracture, potential influence of fat emboli and mobilized mesenchymal stem cells. Polytrauma may produce the added risk of central influences of sepsis, SIRS and hemorrhagic shock

## Traumatic injuries

Traumatic injuries are physical insults to the body of sudden onset that are induced by mechanical forces and commonly occur in motor vehicle accidents, slips and falls, sports activities, industrial accidents, assaults, and the warzone [1–6]. Trauma injuries accounted for 5.8 million deaths globally in 1998, and this number is estimated to increase to 8.4 million annual deaths by 2020 [7]. In the USA, each year, approximately 30 million individuals are hospitalised from injuries sustained from motor vehicle accidents, slips and falls, or being struck by/against an object, with the estimated lifetime medical costs associated with these injuries thought to be over $50 billion [8]. Traumatic injuries are commonly classified and quantified using the Abbreviated Injury Scale (AIS) [9]. The AIS is an anatomical-based trauma scoring system that is used to evaluate and rank injuries on a numerical scale ranging from 1 (mild; e.g. superficial laceration) to 6 (fatal injuries) in nine independent body regions, i.e. the head, face, neck, thorax, abdomen, spine, upper extremity, lower extremity, and external regions [9]. Of particular relevance to TBI, the Glasgow Coma Scale (GCS) is the universally accepted severity classification system for TBI. The GCS is comprised of three component tests involving the Eyes, Motor, and Verbal scales. The Eyes scale ranges from 1 (no response) to 4 (spontaneous). The Motor scale ranges from 1 (no response) to 6 (obeys commands), and the Verbal scale ranges from 1 (no response) to 5 (oriented and converses normally). The summation of these scales allows for a total GCS score ranging from 3 to 15. Based on these scores, the GCS classifies TBI cases as mild (GCS 14–15), moderate (GCS 9–13), or severe (GCS 3–8).

## Multitrauma and polytrauma

Due to the high-impact nature of trauma-inducing accidents, it is common for patients to suffer injuries to multiple body regions [5, 6]. The simultaneous traumatic injury to multiple body regions has traditionally been labelled as multitrauma or polytrauma, with the terms being used interchangeably. However, in recent years, there have been significant efforts to establish an international consensus regarding the specific definitions of polytrauma and multitrauma. In 2014, an expert panel proposed that the term polytrauma be applied when there are two injuries that are AIS ≥ 3 and one or more additional diagnoses (pathologic condition), that is, hypotension (systolic blood pressure ≤90 mmHg,), unconsciousness (GCS score ≤8), acidosis (base deficit ≤−6.0), coagulopathy (partial thromboplastin time ≥40 s or international normalized ratio ≥1.4), and age (≥70 years) [10]. Alternatively, it has been proposed that multitrauma be defined as injury to more than one body region (not exceeding AIS ≥ 3 in two regions) without systemic inflammatory response syndrome (SIRS) [11]. Because these are relatively new definitions, they have not yet been adopted worldwide, and previous studies in the field often use vague and inconsistent terminology. Therefore, in this review, the term polytrauma will only be applied when the specific criteria defined above by Pape and colleagues [10] is clearly met, whereas multitrauma will be more broadly defined as traumatic injury to multiple body regions (which may or may not encompass polytrauma).

According to the Trauma Audit and Research Network (TARN) database, multitrauma involving significant head injury results in the highest risk of mortality amongst all multitrauma patients and of all head injury patients, around one third are deemed to have suffered multitrauma [12]. Importantly, the occurrence of multitrauma often creates significant challenges for medical professionals when compared with monotrauma (i.e., injury to one body region), largely due to the potential for combined and interactive pathophysiologies between different organ systems. [11] In particular, the multitrauma patient with TBI may be at greater risk of suffering adverse effects of such combined and/or interactive processes, particularly given isolated TBI itself is a condition with an inherent complex pathophysiology and high risk for disability or mortality [13]. Herein, we review the literature on multitrauma involving TBI with concomitant peripheral injuries, with a particular focus on how extracranial injuries may affect TBI pathobiology and outcome.

## Traumatic brain injury

TBI induced by mechanical forces is a spectrum disorder currently classified as mild (e.g., concussion), moderate, and severe. It is estimated that over 10 million people suffer from TBI each year worldwide [14], and compared to other types of traumatic injuries, it is the most common to result in death or disabilities [15, 16]. In the USA alone, approximately 1.7 million people sustain a TBI annually, with nearly 80 % treated in emergency, 16 % hospitalised, and 3 % resulting in death [17]. Those who survive the initial TBI insult are often left with long-term neurological deficits and a general decrease in quality of life. Outcomes from TBI can be temporary or permanent dysfunction of cognition, motor function, physiology, and psychology [13] and post-TBI neurological disorders, such as chronic traumatic encephalopathy (CTE), dementia, and posttraumatic epilepsy [18, 19]. All of the abovementioned factors contribute to the large economic burden of TBI, with direct costs per TBI case approximately range from $25,000 to $114,000 according to studies in the USA [14].

Brain damage occurring in TBI results from either primary or secondary injury mechanisms. Primary injury involves tissue damage caused directly by mechanical forces at the moment of impact and may include blood-brain barrier disruption, necrosis, and axonal shearing [13, 16, 20]. The primary injury alone can lead to intracranial haemorrhage, cerebral oedema, and hydrocephalus [13, 16, 20]. The progressive brain damage is due to a number of secondary injury processes including neuroinflammation, oxidative stress, apoptosis, metabolic abnormalities, proteopathies, and further injury to the blood-brain barrier and axons [13, 16, 20]. Secondary injury processes may occur within minutes to hours after TBI [21–23] and, depending on the severity and condition, may persist and progress into chronic neurodegeneration [13]. To date, however, the multifaceted pathophysiology of TBI is not yet fully understood. Consequently, all phase III clinical trials have failed and currently no treatment that is known to improve long-term neurological outcomes in TBI patients. Importantly, all of the drugs showed significant improvement in positive outcomes in various pre-clinical TBI models, and all pre-clinical trials used clean, monotrauma head injury models. These translational failures may be in part due to researchers and clinicians overlooking important and common factors that may affect TBI pathobiology, such as concomitant extracranial injuries.

## Potential influence of peripheral injury on TBI pathobiology and outcome

Blood-brain barrier disruption in TBI provides unprecedented access for peripheral factors to enter the normally immune-privileged brain parenchyma. Several studies have demonstrated the migration of peripheral leukocytes into injured brain tissue following TBI [24–26]. However, in multitrauma patients with TBI and concomitant extracranial injury, the potential for peripheral involvement in the pathobiology of TBI is substantially greater than for patients with isolated TBI. For example, peripheral injuries involving significant inflammatory responses may have the potential to exacerbate the neuroinflammatory response of TBI. The neuroinflammatory cascade in TBI is characterized by the activation of the brain’s microglia and astrocytes, the release of inflammatory cytokines by neurons and glia [27, 28]. Peripheral injuries such as bone fracture can increase circulating levels of many of the inflammatory cytokines that mediate TBI pathobiology [29–33]; therefore, the multi-injured patient may be at increased risk of suffering from worsened TBI outcomes. In addition, significant extracranial injuries have the potential to induce other systemic changes, such as haemorrhagic shock [34, 35], fat embolism [36, 37], and elevated levels of reactive oxygen species [38] and growth factors [29, 39, 40], all of which have the potential to influence the secondary injury process of TBI. However, despite the high prevalence of multitrauma, and an increasing recognition of potential interactions in injury pathologies, multitrauma involving TBI remains poorly understood and is under studied both in the experimental and clinical settings [41]. Recently, however, several clinical and basic investigations have emerged describing influences of peripheral injuries on TBI pathobiology and outcomes, indicating a greater appreciation of the complexities of multitrauma may allow for more appropriate care in this subset of TBI patients.

## Clinical multitrauma/polytrauma investigations

Several clinical studies have investigated the effect of concomitant extracranial injuries on outcomes following TBI, though the findings of these studies have been mixed. While many have reported increases in patient mortality [15, 42–45], worsened functional outcomes [43, 46, 47], and other effects [27, 48, 49], some have concluded that the presence of concomitant extracranial injury has little to no influence on TBI outcome [50–52]. Because the vast majority of clinical multitrauma and polytrauma studies have been conducted retrospectively using trauma databases, interpretations of these studies must be tempered by the existence of several limitations and confounding variables. Many of these variables are difficult to avoid in the clinical setting, such as variations in injury locus and mechanism and differences in treatments (pharmacological or surgical). Further confounding multitrauma and polytrauma investigations is that the average age of multi-injured patients is often significantly lower than for isolated TBI patients ([43, 48, 51, 53], see Table 1—limitations). There are linear relationships between increasing age and both unfavourable outcomes and mortality in adult TBI patients [54]. Consequently, the impact of multitrauma or polytrauma on TBI outcomes may be masked by improved outcomes related to the younger age of these patients, particularly when compared to older isolated TBI patients. Additionally, a significant limitation in the design of many of these studies lies in the variable, often ambiguous definition and separation of injury location and severity. Though many have utilized the conventional GCS and AIS scores to define injury severity upon admission, most fail to separate results based on extracranial injury location and the various combinations of GCS and AIS scores. Furthermore, these injury severity scales are highly objective and do not account for mechanism of injury. Perhaps the most significant factor limiting previous clinical investigations into the influence of peripheral injury on TBI outcomes is the restricted nature of the assessed outcomes, with nearly all studies conducted retrospectively and therefore primarily analysing mortality rates and/or Glasgow Outcome Scale (GOS) available in databases such as the Corticosteroid Randomisation After Significant Head Injury (CRASH) and International Mission for Prognosis and Analysis of Clinical Trials in TBI (IMPACT). Though these investigations fail to reveal the specific influence peripheral injury may have on the multifaceted neurological impairments associated with TBI, particularly given the GOS has been criticized for its lack of sensitivity and may itself be directly influenced by concomitant injuries, overall, they do suggest a possible association between extracranial injuries and worsened patient outcome post-TBI (Table 1).

**Table 1:** Clinical studies investigating the effect of extracranial injury (ECI) on traumatic brain injury (TBI)

Studies reporting effects of ECI on TBI			
Author	Subjects	Major Relevant Findings	Limitations
Kumar et al., 2015	114 TBI patients TBI: GCS ≤ 8, AIS ≥ 3 and evidence of damage by CT PT: TBI + AIS ≥ 3 at least one ECI	- Weekly average patient serum IL-6 and IL-10 higher in PT compared to isolated TBI group - No difference in GOS scores between TBI and PT patients	–TBI group older than PT (Mean age 39 v 30) –PT group higher incidence of skull fractures –Low numbers
Leitgeb et al., 2013	767 TBI patients TBI: AIS 2–5 (Mean 4) MT: TBI + AIS > 2 at least one ECI	–Mortality higher in mild TBI (AIS = 2) patients with ECI, however overall mortality comparable between groups –Favourable outcome (GOS 4/5) reported in more MT patients	–TBI group older than MT (Mean age 61 v 46) –Orthopaedic interventions common (25% MT had ECI surgery) but not accounted for
Lingsma et al., 2013	508 TBI patients TBI: GCS 9–13 (moderate), 3–8 (severe) MT: TBI + ECI (Median ISS = 4)	–Unfavourable outcome (GOS = 2/3) and mortality higher for patients with moderate TBI and ECI than for isolated moderate TBI	–Missing patients in follow up –Variability in ECI severity
Leong et al., 2013	100 TBI patients TBI: mild (GCS 13–15), moderate (9–12), severe (3–8) MT: TBI + AIS ≥ 3 at least one ECI	–Fewer mild TBI patients with ECI made ‘good recovery’ (GOS = 5) compared with those with isolated mTBI	–Low numbers
Van Leeuwen et al., 2012	39,274 TBI patients (IMPACT, CRASH, TARN databases) TBI: GCS 13–15 (mild), 9–12 (moderate), 3–8 (severe) MT: TBI + AIS ≥ 3 (or requiring hospital admission) at least one ECI	–IMPACT/CRASH pooled adjusted OR: Effect of ECI on mortality = 2.14 in mild-, 1.46 moderate-, 1.18 severe-TBI. –TARN adjusted OR higher in MT patients (2.81 mild-, 2.18 moderate-, 2.14 severe-TBI).	–Missing values in databases –Variability in ECI severity –No functional outcomes
Lefering et al., 2008	21,356 trauma patients Various combinations of TBI and/or ECI to extremity or torso (AIS 0–6)	–Non-significant increase in mortality in MT versus TBI only overall, but significant difference at all TBI severities when ECI AIS ≥ 4	–No GCS used –No functional outcomes
Hensler et al., 2002	125 trauma patients TBI: GCS ≤ 8 or AIS ≥ 3 PT with TBI: TBI + AIS ≥ 3 for one ECI PT without TBI: AIS ≥ 3 for two ECI	–Serum levels of IL-6, IL-10, 55- and 75-kDa soluble tumor necrosis factor receptors, polymorphonuclear neutrophil elastase all lower in patients with TBI only compared with PT (with and without TBI)	–Low numbers –TBI group older than PT (Mean age 42 v 36) –Only pathological changes reported
Siegel et al., 1991	1709 TBI patients TBI: GCS 13–14 (mild), 9–12 (moderate), 7–8 (moderately severe), 4–6 (severe), 3 (near fatal) MT: TBI + ECI separated by location (no defined AIS)	–Overall mortality rate (all GCS) almost tripled in blunt TBI patients with pelvis or femur fracture (similar results for lung-, liver-, bowel-, major vessel- injury). –Increase in mortality most substantial in mild to moderate TBI.	–Variability in ECI severity –No functional outcomes
Gennarelli et al., 1989	16,524 TBI patients TBI: AIS 2–6 MT: TBI + AIS 1–6 at least one ECI	–Synergistic effect on mortality when moderate head injuries (AIS 3–4) combined with severe ECI (AIS 4–6). –No additive effect on mortality when ECI AIS ≤ 3	–No GCS used
Studies reporting no/inconclusive effects of ECI on TBI			
Author	Subjects	Major Relevant Findings	Limitations
Stulemeijer et al., 2006	299 TBI patients TBI: GCS 13–15 MT: TBI + AIS ≥ 2 for one ECI	–Mild TBI patients with ECI had worsened GOS-E scores, however no differences in post-concussion symptoms at 6 months –Acute symptoms (headache, dizziness, nausea) lower in MT group	–TBI slightly more severe in MT patients –Symptoms masked by frequent use of analgesic in MT patients?
Sarrafzadech et al., 2001	119 trauma patients TBI: GCS 3–7, Mean total ISS = 25 MT: TBI + AIS other 1–6, Mean total ISS = 39.5 ECI only: AIS other 1–6, Mean ISS = 29	–No difference in physiological variables between patients with isolated TBI or MT with TBI between days 1–12 post-injury –No differences in GOS score at 6 and 12 months between TBI and MT patients, higher GOS scores in extracranial injury only patients.	–TBI group older than MT (Mean age 36 v 28) –Low numbers –Wide range of injury severities and locations
Baltas et al., 1998	386 TBI patients TBI: GCS 3–8 MT: TBI + ECI (no defined AIS)	–No difference in mortality in MT versus TBI only groups.	–No defined AIS for extracranial injury –No functional outcomes

*ECI* extracranial injury, *MT* multitrauma, *PT* polytrauma

**Table 2:** Animal studies on the effects of extracranial injury on TBI

Author	Subjects	Major Relevant Findings	Limitations
Yang et al., 2016	Male C57BL/6 mice. 12–14 weeks old Sham: Incisions etc., no trauma TBI: CCI (open skull, 4.5 m/s, penetration depth 1 mm) MT: TBI + FX (Tibia, intramedullary pin) MT treated: TBI+ HMGB1 (10mg/kg 60 min prior to FX)	–Elevated brain levels of IL-6 at 2- and 4-days in MT mice compared to TBI mice, higher levels of TNF-α and IL-1β levels at 4 days. –Brain lesion volume and edema elevated in MT mice compared to TBI mice at 4 days –MT mice pre-treated with HMGB1 had reduced neurological scores, edema and brain lesion volumes at 2 and 4 days	–No FX only group: FX affect neurological scores? –No neuroinflammation analysis following HMGB1 treatment –Acute analysis only –Confounding craniotomy
Shultz et al., 2015	124 male C57BL/6 mice. 12 weeks old Sham: Incisions etc., no trauma TBI: Weight-drop (333 g rod, 2 cm drop) FX: Tibia (intramedullary pin) MT: TBI + FX	–Brain IL-1β levels higher in MT group compared to all groups at 24 h and 35 days, GFAP elevated in MT mice at 24 h and 35 days, neutrophil highest in MT mice at 24 h –Edema and blood–brain barrier damage higher in MT group compared to all groups at 24 h –Lateral ventricle enlargement and diffusion abnormalities in MT mice not found other groups at 35 days –Altered anxiety-related behavior in MT mice compared to all groups at 35 days	- Variability in serum cytokine levels –No chronic time-point
Weckbach et al., 2013	Male C57BL/6 mice. 8–9 weeks old. Sham: Incisions etc., no trauma Blunt chest trauma (ChT): Blast wave to thorax TBI: Weight-drop (333 g rod, 2 cm drop) FX: Femur (intramedullary pin) with soft tissue injury MT (3 groups): TBI + ChT or FX + ChT or TBI + FX PT: TBI + ChT + FX	–Serum IL-6 higher in PT mice compared to all other groups at 2 h, only elevated in PT and MT mice involving TBI at 6 h –Serum G-CSF and CCL-2 higher in MT mice with TBI than for TBI only mice –Serum neutrophil apoptosis marker expression decreased in PT animals only	–Only acute time-point analysis –Systemic analysis only
Probst et al., 2012	45 male C57BL/6 mice. 8–10 weeks old. TBI: Weight-drop (3m/s; weight details not included) FX/Shock: Femur (wood splint) + 60% blood vol. loss PT: TBI + FX/Shock	–Mortality rates higher in PT compared to FX/Shock and TBI only –Serum IL-6, TNF-α and CCL-2 higher in PT animals compared to FX/Shock and TBI only animals at 4 days	–No sham/control animals –Single time-point –Systemic analysis only
Weckbach et al., 2012	352 male Wistar rats, 10–12 weeks old Sham: Incisions etc., no trauma Blunt chest trauma (ChT): Blast wave to thorax TBI: Weight-drop (severity not disclosed) FX: Tibia-fibula (fixation not disclosed) + soft tissue injury MT (2 groups): TBI + ChT or ChT + FX PT: TBI + ChT + FX	–Serum IL-6 singificantly increased in PT animals only –Serum neutrophil chemoattractant expression elevated only in multiply injured animals –No changes in serum TNF-α	–Only acute time-point analysis –No FX only group –Variability in serum cytokine levels –Systemic analysis only
Mirzayan et al., 2012	60 male C57BL/6 mice. 8–10 weeks old. TBI: CCI (open skull, 3 m/s, penetration depth 1 mm) FX/Shock: Femur (un-supported) + 60% blood vol. loss PT: CCI + FX/Shock	–Trend (*p* = 0.068) towards elevated reactive astrocyte (GFAP) density in the ipsilateral hippocampus of PT compared to TBI only mice at 4 days	–No sham animals (only controls)- –Single time-point analysis
Maegele et al., 2007	100 male Sprague–Dawley rats, 300–250g Control: No trauma TBI: LFP (2.1 atm) FX: Tibia (un-supported) MT: TBI + FX	–Serum IL-6 and IL-10 levels higher in MT rats compared TBI only and FX only rats during first week post-injury –No differences in serum tumour necrosis factor receptor 1 and IL-1β between groups	–Small numbers per group for plasma analysis (3–5) –No sham animals (only controls) –Systemic analysis only –Confounding craniotomy

*MT* multitrauma, *PT* polytrauma, *FX* fracture

### Patient mortality

The most widely studied outcome in clinical investigations of multitrauma/polytrauma with TBI is mortality rate of patients surviving the early stage (first hours) of injury. Several studies have provided evidence for potential synergistic effect of concomitant head and peripheral injuries on mortality. The most comprehensive study to date on the influence of concomitant extracranial injuries on mortality rates in TBI patients is a meta-analysis of the IMPACT, CRASH, and TARN databases conducted by van Leeuwen and colleagues [45]. By compiling a pooled, odds ratio adjusted for core prognostic parameters (e.g. age, GCS motor score, pupil reactivity, hypotension), this large-scale observational study provides strong evidence that a major extracranial injury (AIS ≥ 3) increases mortality rates in TBI patients [45]. However, it is important to note that extracranial injuries had less impact on outcomes of patients with severe TBI (compared with that for mild and moderate TBI patients).

This conclusion drawn from this meta-analysis is similar to that made by other large-scale trauma registry studies, reporting significant effects of peripheral injury on mortality in mild to moderate TBI, but not in severe TBI. Genarelli and colleagues [15] found that mortality following TBI was not influenced by extracranial injuries, except when moderate head injuries (AIS 3–4) were accompanied with very severe extracranial injuries (AIS 4–6) [15]. A recent Austrian study also reported similar mortality rates between isolated TBI (AIS head <6) and TBI plus concomitant peripheral injury (AIS > 2); however, for patients with a mild TBI (AIS head = 2), mortality rate was significantly higher in those also suffering peripheral injury [43]. Another recent study used the IMPACT prognostic model to assess the influence of extracranial injury on TBI and confirmed that concomitant peripheral injury was associated with increased mortality in patients with mild-moderate (GCS 9–13) but not with severe TBI (GCS 3–8) [47]. Siegel and colleagues [44] found mortality rate was considerably higher in patients with extracranial injuries (AIS ≥ 3) and blunt TBI of all severities (GCS 3–14); however, the most substantial increase in mortality rate was observed in multitrauma patients with mild-moderate TBI (GCS 13–15, 9–12) [44]. A study of severely head injured patients (GCS 3–8) found no difference in mortality between isolated TBI and TBI with extracranial injuries (no defined AIS), again suggesting that additional injuries likely have little influence on mortality following severe TBI [50]. Lefering et al. [42] however found that extracranial injuries of AIS grade 4 and above significantly increased TBI mortality across a whole range of TBI severities (AIS 1–6) [42].

The aforementioned clinical studies have several caveats, including wide range of ages of patients enrolled in the studies [43, 51], the use of different injury classification schemes [15, 43, 47, 50], and the use of the inferior AIS rather than GCS scores to classify TBI severity [15, 42, 43]. Furthermore, the majority of the mortality-focused multitrauma studies did not feature an extracranial only (without TBI) group [43–45, 47, 50], and all failed to report cause of death. These shortcomings make it difficult to understand the effect of concomitant injuries on TBI-related mortality. Despite these limitations, taken together, these clinical findings provide some evidence of a correlation between presence of concomitant extracranial injuries and increased risk of mortality, particularly following mild-moderate TBI. Further work is needed however to establish whether this possible increased risk of death is largely a direct consequence of peripheral injury or the result of these injuries potentiating TBI pathobiology.

### Functional outcomes

Assessments of functional outcomes of multitrauma/polytrauma patients with TBI are confined to a small number of studies that have conducted basic functional testing in the months following injury. Functional outcomes findings of these studies are mixed, with recovery predominately assessed using the GOS or GOS-Extended (GOS-E). The GOS rates patient recovery on a five-category scale, i.e. death, vegetative state, severe disability, moderated disability, or good recovery. The GOS-E further categorizes recovery into eight categories by subdividing categories of severe disability, moderated disability and good recovery into a lower and upper category. Kumar et al [53] found no difference in GOS scores at 6 months post-injury between severe TBI (AIS head ≥3) and polytrauma patients (AIS head ≥3, GCS ≤ 8, AIS other > 3). These findings were likely compromised by the increased mean age of isolated TBI patients [53], with increased age throughout adulthood associated with higher likelihood of unfavourable outcome post-TBI [54]. This same age difference was also a likely confounding factor in the study by Letigeb and co-workers [43], who surprisingly reported favourable outcomes at 6 months in significantly more multitrauma patients (AIS head 1–6, AIS other >2) than patients with TBI only (AIS head 1–6). As with the previously discussed mortality data, it is tempting to speculate on a possible correlation between TBI severity and the potential for extracranial impact on functional recovery, as some researchers have described worsened functional outcomes in mild TBI patients. Recently, a study by Lingsma et al. [47] reported that functional outcome (GOS-E) was primarily affected by extracranial injuries following mild-moderate (GCS 9–13) but not severe TBI (GCS 3–8). Another study on patients with mild-TBI (GCS 13–15) found worsened GOS scores at 18 months post-injury in patients with concomitant injuries (AIS ≥ 3) [46]. Similarly, a study on patients with either mild-TBI (GCS 13–15) and mild-TBI with extracranial injuries (AIS ≥ 2) found those with additional injuries had worsened GOS-E scores at 6 months post-injury; however, they reported no differences in post-concussion symptoms at this time-point between groups [52]. Though further investigations are essential, these studies do suggest that functional outcome after TBI is also impacted by concomitant injuries.

The conclusions of these studies are not only limited by the confounding variables discussed earlier but also by the nature and timing of the functional assessments. The GOS and GOS-E assessments are still the most commonly used primary outcomes for assessing patient recovery [55, 56]; however, the insensitivity and subjective nature of these assessments are often criticized and now recognized as a factor possibly contributing to the failure of several TBI clinical trials [57, 58]. There is a growing consensus that improved functional assessments will require use of outcome measures that better quantify, without subjectivity, the many aspects of deficits associated with TBI, such as cognitive, sensory, motor, and emotional function [57, 58]. Furthermore, inferences on the effect of multitrauma/polytrauma on TBI may be confounded by the likely direct effect of major, persisting extracranial injuries have on GOS scores. Finally, the conclusions of these studies are limited by the single, 6-month end-point chosen for nearly all GOS assessments. Given TBI is a highly heterogeneous condition that often features chronic and progressive neurodegeneration, development of longitudinal studies with repeated functional assessments will enable greater characterization of short- and long-term deficits that may be associated with concomitant extracranial injuries and TBI.

### Pathobiology

The injured brain is more susceptible to secondary insults, such as ischemia [59], hypotension [60], sepsis [61], seizures [62], or surgical interventions albeit very few clinical studies have investigated the effect of these insults on neuroinflammation and other pathobiological process of TBI. The effect of extracranial injuries on TBI pathobiology and thus outcome is demonstrated by the contentious issue of bone fracture fixation procedures in TBI patients. Many clinics practice ‘damage control orthopaedics’ and delayed definitive fracture fixation to minimize ‘secondary hits’ to the injured brain [37, 63, 64]. While these orthopaedic practices are hypothesized to decrease the risk of worsened brain injury outcome by reducing the potential for both haemodynamic complications and an exacerbated inflammatory response, it is still debated whether these trauma practices improve outcome for TBI patients and, indeed, that these ‘secondary hits’ actually influence TBI pathobiology [64].

The systemic effects of isolated TBI and general trauma have been widely investigated and are beyond the scope of this review (see Keel and Trentz 2000 for review [35]); however, the findings of these trauma studies may have important implications when considering the potential influence of extracranial injury on TBI. Of particular, relevance here is the trauma-induced development of SIRS, a condition in which the extensive inflammatory state often leads to damage of non-injured organs, such as the lung, liver, and kidney [35, 63, 65, 66]. Given TBI frequently features extensive damage to the blood-brain barrier [67, 68], the normally immune-privileged brain tissue becomes vulnerable to secondary insults from the periphery [69]; therefore, it could be speculated that polytrauma-induced SIRS may also influence the brain parenchyma, potentially exacerbating the neuroinflammatory response and worsening brain injury. As with many aspects of clinical research however, such mechanistic understandings are difficult to clearly establish, particularly given the already complex and variable nature of secondary brain injury.

Trauma is strongly associated with SIRS development, and multiple traumas are thought to increase risk of SIRS [35], but surprisingly few clinical studies have characterized the systemic effects of TBI combined with extracranial injury. Studies on patients with severe TBI (GCS ≤ 8) found those that also had a major extracranial injury (AIS ≥ 3) had significantly higher serum levels of pro-inflammatory cytokine interleukin-6 (IL-6) during the first week post-injury [48, 53]. Hergenroeder et al. [70] demonstrated a correlation between serum IL-6 and intracranial pressure was present in patients with isolated severe TBI. However, this correlation was not seen in multiply injured patients, with the authors suggesting orthopaedic trauma-induced increases in serum IL-6 reduced the prognostic accuracy of serum IL-6 in predicting intracranial pressure [70]. Interestingly, serum levels of the anti-inflammatory cytokine interleukin-10 (IL-10) are also elevated in patients with both severe TBI and extracranial injury when compared with isolated TBI only [48, 53], suggesting multitrauma may also feature heightened expression of anti-inflammatory factors.

Other inflammatory cytokines such as interleukin-1β (IL-1β), tumour necrosis factor-α (TNF-α), and granulocyte-macrophage colony-stimulating factor (GM-CSF) have been shown to be elevated in the systemic circulation following peripheral injury (e.g. bone fracture) [29, 31–33, 71] as well as in TBI [27, 72, 73]. It is therefore likely that multitrauma patients may too have elevated post-injury serum levels of these cytokines, but to the best of our knowledge, this has not been investigated. Given IL-1β, TNF-α, and GM-CSF are implicated in TBI [27, 72, 74–76], analysis of serum and cerebrospinal fluid (CSF) levels of these cytokines may provide important insights in to the pathobiology of TBI following multitrauma.

Serum levels of S-100B, the protein biomarker frequently used in pre-clinical and also in clinical TBI studies [72], has also been measured in the serum of multitrauma patients. S-100B can be of extracranial origin, potentially reducing its suitability as a serum biomarker for TBI severity. In an attempt to determine the influence of extracranial injuries on S100B expression, Savola and colleagues analysed serum of patients with isolated TBI, isolated extracranial injuries, and combined injuries [49]. This study found median serum levels of S100B correlated with TBI severity (isolated mild TBI 0.15 μg/L, isolated moderate to severe TBI 0.94 μg/L). While they found isolated large extracranial injuries (e.g. large fractures) moderately increased serum levels (0.35 μg/L), they also found that when combined with brain injury, large extracranial injuries substantially increased circulating S100B levels in both mild (0.93 μg/L) and severe (4.01 μg/L) TBI patients [49]. The moderately elevated expression of S100B for isolated large extracranial injures led the authors to conclude S100B may not be a suitable predictive marker for TBI in multitrauma patients [49]. Nonetheless, the substantial increase in S100B levels observed in patients with both injury types could be interpreted as possible evidence of peripheral and central injury interactions or common pathomechanisms. Further investigations are clearly necessary to support such speculation.

In summary, while clinical data is insufficient, particularly on the influence of multitrauma on the secondary injury process after TBI, the aforementioned serum data provides preliminary evidence that multitrauma may lead to systemic inflammatory changes that have the potential to affect the neuroinflammatory response of TBI. Future clinical studies with clearly defined, specific research questions (e.g. how does limb fracture alter inflammatory profiles of patients with mild TBI?) are required to unearth the particular pathological consequences and possible therapeutic implications for multiply injured patients.

## Animal studies

Because clinical multitrauma and polytrauma are highly heterogeneous conditions that can involve various combinations of body regions and injury severities, it is not possible to have a single animal model that is representative of all multitrauma/polytrauma scenarios. However, the criteria used to define clinical multitrauma and polytrauma (described above), and clinical studies identifying the most common trauma injury combinations have provided some guidance for developing initial animal models with a high degree of clinical relevance. Rodent models of multitrauma/polytrauma featuring TBI have been developed over the last 10 years, enabling insights into the influence of multiple injuries on both the systemic and central inflammatory response, as well as preliminary analysis on the structural and functional TBI outcomes (Table 2). Importantly, pre-clinical studies to date have incorporated the two most common forms of significant extracranial injury in TBI patients, namely TBI combined with extremity fracture or TBI combined with thoracic trauma [6]. Herein, we discuss the recent pre-clinical progress in both multitrauma and polytrauma studies involving TBI.

In the initial experimental multitrauma studies featuring rats, TBI was induced by lateral fluid percussion injury (LFP) immediately followed by a closed tibial fracture [77, 78]. These preliminary studies found that circulating levels of IL-6 and IL-10 were significantly elevated in the first 24–48 h following injury in animals with combined injury compared to isolated injury only. However, the non-stabilized tibial fracture impaired hind limb function and thus compromised the assessment of locomotor function between groups [77, 78]. Nonetheless, the use of this novel multitrauma model showed for the first time that the systemic inflammatory response might be heightened in animals with both TBI and significant peripheral injury.

Intensified systemic inflammatory responses have since been reported in a number of subsequent animal models of combined TBI and peripheral injury. In a murine polytrauma model featuring weight-drop TBI along with femoral fracture followed immediately by haemorrhagic shock, circulating levels of IL-6, TNF-α, and chemokine CC ligand-2 (CCL-2) were substantially elevated at 4 days post-injury in animals with combined injury compared to isolated injuries only [79]. Increased systemic inflammation was also reported in rats with combined TBI, chest trauma, and tibial/fibular fracture, with circulating IL-6 significantly elevated in polytraumatized animals but not those with isolated injuries only at 4 h post-injury [80]. A subsequent similar investigation in mice found that animals with TBI, bone fracture, and chest trauma had elevated serum levels of IL-6 compared to all other groups at 2 h post-injury, and by 6 h post-injury, IL-6 expression was only elevated in serum of multiply injured animals involving TBI [81]. This same study also found that compared to mice with TBI only, those given TBI along with femoral fracture had increased circulating levels of granulocyte colony-stimulating factor (G-CSF) and those given a TBI and chest trauma had elevated plasma CCL-2 concentrations at 6 hours post-injury [81]. Furthermore, expression of markers of neutrophil apoptosis at 2 and 6 h post-injury were markedly decreased in animals with three injuries (TBI, fracture, and chest injury), but not animals with single or double injuries. Interestingly, many of the aforementioned increases in systemic inflammation in experimental multitrauma/polytrauma studies may not simply be an additive effect of the combined injuries, as levels of several circulating inflammatory markers appear to be significantly elevated when compared to the summated responses for the isolated injuries. Taken together, these basic trauma findings provide some evidence that multiple traumas can alter the systemic immune-inflammatory response post-injury. The potential for such alterations to impact peripheral organs is beyond the scope of this review; the remainder of this review will discuss the preliminary animal-based evidence suggesting a potential impact of peripheral trauma on the pathological changes of the injured brain.

The potential for peripheral injury affecting TBI outcomes is supported by a number of studies providing evidence that peripheral immune factors can alter experimental TBI pathology. A systemic injection of lipopolysaccharide in rats with a cortical contusion injury was shown to increase the injury site expression of inflammatory and apoptosis markers compared to vehicle-treated rats with TBI [82]. Furthermore, peripheral administration of IL-1β in rats with moderate LFP was shown to significantly increase structural and behavioural deficits compared to vehicle-treated animals, possibly due to a heightened neuroinflammatory response in IL-1β treated rats [83]. In addition, a recent study found treatment with an antibody targeting leukocyte infiltration significantly reduced neuroinflammation and behavioural deficits in rats with severe TBI [26], indicating the role of peripheral leukocytes in TBI is likely significant.

Despite the evidence that peripheral injuries induced increased systemic inflammatory responses in rodents TBI studies, and the evidence that peripheral immune factors influence TBI, until recently, no studies had compared the secondary injury process (or pathology) of TBI between animals with isolated brain injury and those also with peripheral trauma. Preliminary evidence for altered TBI pathobiology in experimental polytrauma came from mouse model featuring controlled cortical impact (CCI) combined with femoral fracture with hemorrhagic shock, with the investigators reporting a near significant trend towards elevated reactive astrocyte density in the ipsilateral hippocampus of polytrauma mice compared to CCI only mice at 4 days post-injury [84]. The neuroinflammatory response to multitrauma was more extensively analysed in a mouse model featuring weight-drop TBI (mild to moderate severity) and tibial fracture [85]. In this study, mice with tibial fracture had an increased and prolonged neuroinflammatory response, evidenced by the elevated brain tissue concentrations of IL-1β and glial fibrillary acidic protein (GFAP; an indicator of astrogliosis) at 24 h and 35 days post-injury, as well as elevated neutrophil expression, edema, and blood-brain barrier disruption at 24 h post-injury [85]. Furthermore, at 35 days post-injury, magnetic resonance imaging (MRI) showed that multitrauma mice had enlarged ventricles and diffusion abnormalities not seen in mice with isolated TBI, and behavioural testing revealed changes in anxiety-related behaviours in multitrauma mice only. Taken together, the findings demonstrated for the first time that concurrent bone fracture and brain injury can exacerbate structural and behavioural deficits, possibly due to a heightened and prolonged neuroinflammatory response compared with isolated TBI only [85].

An exacerbated neuroinflammatory response to multitrauma was also found in a subsequent study on mice exposed to CCI and tibial fracture [86]. In this model, Yang and colleagues [86] found that when compared to mice with TBI only, mice with TBI and bone fracture had increased brain tissue IL-1β, TNFα, and IL-6 at 4 days post-injury [86]. Furthermore, multitrauma mice also had increased edema and brain lesion volumes compared to mice with TBI only. Neurological severity scores were elevated in multitrauma mice compared to TBI only mice at 4 days post-injury; however, given this study lacked a fracture-only group, it is impossible to rule out the contribution of bone fracture to this finding. Finally, when the authors injected antibodies to the inflammatory mediator high mobility group 1 (HMGB1) at 60 min prior to fracture, they found that compared with untreated multitrauma mice, those treated with neutralizing antibodies to HMGB1 had reduced neurological severity scores and reduced brain damage at 24 and 48 h post-injury. Similar findings were also recently described in a murine model of stroke combined with bone fracture, with mice undergoing bone fracture 24 h after ischemic injury found to have a significantly heightened neuroinflammatory response that was prevented with HMGB1 antibody treatment [87, 88].

The last decade has seen a substantial increase in the number of animal-based studies of multitrauma, resulting in growing body of evidence supporting the hypothesis that multiple injuries may have pathological consequences not seen with isolated injuries. In particular, several studies have demonstrated that concomitant TBI and limb fracture can produce significantly elevations in circulating pro-inflammatory cytokines, with recent findings also suggesting bone fracture may exacerbate the neuroinflammatory response and worsen structural and functional deficits following TBI. Additional studies are however required, both to further characterize the pathobiological consequences of TBI combined with bone fracture and to investigate the possible central influence of other injuries most frequently combined with TBI (i.e. thoracic and/or abdominal injury). When choosing or developing animal models of multitrauma or polytrauma, it is important that researchers place priority on replicating the fundamental clinical characteristics of these conditions, while minimizing the presence of potentially confounding variables. With regard to TBI, it is essential that surgery duration is minimized in order to ensure multiple injuries are able to be delivered in short succession if not simultaneously and to reduce the impact of potentially confounding anaesthesia. In addition, TBI models involving craniotomy (e.g. traditional CCI and fluid percussion injury), a form of bone injury, may also represent a confounding variable. For these reasons, TBI delivered via closed-skull methods such as closed-skull CCI or weight-drop injury, blast injury, or acceleration/deceleration models may avoid confounding craniotomy while still providing high clinical relevance. For investigations into the polytrauma condition, it is recommended that researchers clearly distinguish their model from multitrauma by ensuring it meets the specific criteria outlined for this condition, i.e. presence of two significant injuries (equivalent AIS ≥ 3) along with one or more additional pathological diagnosis (e.g. hemorrhagic shock) [11]. Another factor to consider in attempting to recapitulate clinical trauma is the timing of surgical procedures, with the possible influence of delayed fracture fixation (often performed clinically) on neuroinflammatory profiles and TBI outcomes able to be quantified using animal models. Taken together, animal models of multitrauma and polytrauma should be as representative of the most common features of their clinical counterparts as possible, while still permitting the use of reproducible injuries with minimal presence of confounds. Ultimately, an important question that must be addressed is the generalizability of findings based on common multitrauma and polytrauma combinations to the broader spectrum of these conditions. This is yet another topic that highlights the utility of animal models given the ability to compare different combinations of trauma in highly controlled conditions.

## Future directions

This review has highlighted several lines of evidence suggesting a highly likely influence of extracranial injury on TBI. Nonetheless, despite the recent progress in the area, several significant limitations in the aforementioned studies have prevented the development of a greater understanding of central and peripheral injury interactions following multitrauma, particularly in the clinical setting. Though clinical trauma presents inevitable challenges and several potentially confounding variables, such as heterogeneity of injuries, differences in patient demographics (e.g. age, gender, ethnicity, genetic variances, medical history), and variations in clinical management (e.g. pharmacological and surgical interventions), clinical advances in this area will require greater efforts to implement robust study designs that either control or account for these variables. Furthermore, significant strides in our understanding of TBI in multitrauma/polytrauma patients will require incorporation of clinically relevant outcome measures, such as the various neuroimaging capabilities provided by MRI and positron emission tomography (PET). These methods would allow for in vivo monitoring of brain damage and neuroinflammation in isolated TBI versus TBI patients with extracranial injury [89, 90]. Additionally, nearly all of the clinical investigations to date are limited to single time-point outcome analysis, and given the complex and dynamic nature of TBI pathologies, it is important that future studies consider use of serial longitudinal assessments of various structural and functional outcomes.

Though inflammation seems a likely mechanism of injury interaction in multiply injured patients, to date, no studies have created inflammatory profiles of patients with various combinations and severities of head and peripheral injury. Such characterization will allow researchers to determine not only if inflammation is heightened in multiply injured patients but also the temporal complexities of the inflammatory response, and if certain inflammatory pathways are more affected by particular combinations of multitrauma and therefore more appropriate to target therapeutically. For example, if bone fracture combined with TBI is found to cause a particularly significant increase in IL-1β expression in the early stages post-injury, as found in mice [85], therapies such as IL-1 receptor antagonists may prove to be more effective in this form of TBI. However, if inflammatory responses in multitrauma patients involve generalized hyperinflammation, therapies that target systemic inflammation via neuronal inflammatory reflexes may prove to be more appropriate interventions [91]. Furthermore, the potential contribution of other pathways such as reactive oxygen species [16, 38], fat emboli [36, 37], haemorrhagic shock [34, 60], and mobilized mesenchymal stem cells [92, 93] to TBI pathobiology in the patient with concomitant peripheral injuries are possible but remain unknown (see Fig. 1).

## Conclusions

TBI is a devastating condition that currently lacks a treatment that improves patient outcomes. In light of the many past failures in clinical trials in TBI, we must now recognize and investigate factors that can impact TBI pathophysiology, and ultimately patient outcomes, if we are to one day improve the care of TBI sufferers. Though not without limitations, many of the clinical multitrauma/polytrauma studies discussed in this review indicate that peripheral injuries may increase the risk of mortality and functional deficits following TBI, particularly when severe extracranial injuries are combined with mild to moderate brain injury. In addition, several recent animal studies have provided strong evidence that concomitant injuries may increase both peripheral and central inflammatory responses and that structural and functional deficits associated with TBI may be exacerbated in multiply injured animals. Taken together, the findings of this review suggest that concomitant peripheral injuries are capable of modifying the outcomes and pathobiology of TBI, in particular neuroinflammation, and should be accounted for in future pre-clinical and clinical studies.

## Abbreviations

AIS: Abbreviated Injury Scale
CCI: controlled cortical impact
CCL-2: chemokine CC ligand-2
CRASH: Corticosteroid Randomisation After Significant Head Injury
CTE: chronic traumatic encephalopathy
GCS: Glasgow Coma Scale
G-CSF: granulocyte colony-stimulating factor
GFAP: glial fibrillary acidic protein
GM-CSF: granulocyte-macrophage colony-stimulating factor
GOS: Glasgow Outcome Scale
GOS-E: Glasgow Outcome Scale Extended
HMGB1: high mobility group 1
IL-10: interleukin-10
IL-1β: interleukin-1β
IL-6: interleukin-6
IMPACT: International Mission for Prognosis and Analysis of Clinical Trials in TBI
LFP: lateral fluid percussion injury
MRI: magnetic resonance imaging
PET: positron emission tomography
SIRS: systemic inflammatory response syndrome
TARN: Trauma Audit and Research Network
TBI: traumatic brain injury
TNF-α: tumour necrosis factor-α

## References

[1] Al-Thani H, El-Menyar A, Abdelrahman H, Zarour A, Consunji R, Peralta R (2014). Workplace-related traumatic injuries: insights from a rapidly developing Middle Eastern country. J Environ Public Health.

[2] Dobscha SK, Clark ME, Morasco BJ, Freeman M, Campbell R, Helfand M (2009). Systematic review of the literature on pain in patients with polytrauma including traumatic brain injury. Pain Med.

[3] Gennarelli TA, Champion HR, Copes WS, Sacco WJ (1994). Comparison of mortality, morbidity, and severity of 59,713 head injured patients with 114,447 patients with extracranial injuries. J Trauma.

[4] Krug EG, Sharma GK, Lozano R (2000). The global burden of injuries. Am J Public Health.

[5] MacGregor AJ, Mayo JA, Dougherty AL, Girard PJ, Galarneau MR (2012). Injuries sustained in noncombat motor vehicle accidents during Operation Iraqi Freedom. Injury.

[6] Probst C, Pape HC, Hildebrand F, Regel G, Mahlke L, Giannoudis P (2009). 30 years of polytrauma care: an analysis of the change in strategies and results of 4849 cases treated at a single institution. Injury.

[7] Murray CJ, Lopez AD (1997). Alternative projections of mortality and disability by cause 1990-2020: global burden of disease study. Lancet.

[8] Corso P, Finkelstein E, Miller T, Fiebelkorn I, Zaloshnja E (2006). Incidence and lifetime costs of injuries in the United States. Inj Prev.

[9] Stevenson M, Segui-Gomez M, Lescohier I, Di Scala C, McDonald-Smith G (2001). An overview of the injury severity score and the new injury severity score. Inj Prev.

[10] Pape HC, Lefering R, Butcher N, Peitzman A, Leenen L, Marzi I (2014). The definition of polytrauma revisited: an international consensus process and proposal of the new ‘Berlin definition’. J Trauma Acute Care Surg.

[11] Butcher N, Balogh ZJ (2009). The definition of polytrauma: the need for international consensus. Injury.

[12] Lecky FE, Bouamra O, Woodford M, Alexandrescu R, O’Brien SJ. Epidemiology of polytrauma. In: Pape HC, Peitzman A, Schwab CW, Giannoudis PV, editors. Damage control management in the polytrauma patient. New York: Springer; 2010. p. 13–23.

[13] Blennow K, Hardy J, Zetterberg H (2012). The neuropathology and neurobiology of traumatic brain injury. Neuron.

[14] Humphreys I, Wood RL, Phillips CJ, Macey S (2013). The costs of traumatic brain injury: a literature review. Clinicoecon Outcomes Res.

[15] Gennarelli TA, Champion HR, Sacco WJ, Copes WS, Alves WM (1989). Mortality of patients with head injury and extracranial injury treated in trauma centers. J Trauma.

[16] Werner C, Engelhard K (2007). Pathophysiology of traumatic brain injury. Br J Anaesth.

[17] Faul M, Xu L, Wald MM, Coronado V, Dellinger AM (2010). Traumatic brain injury in the United States: national estimates of prevalence and incidence, 2002-2006. Inj Prev.

[18] Starkstein SE, Jorge R (2005). Dementia after traumatic brain injury. Int Psychogeriatr.

[19] Diamond ML, Ritter AC, Failla MD, Boles JA, Conley YP, Kochanek PM (2014). IL-1beta associations with posttraumatic epilepsy development: a genetics and biomarker cohort study. Epilepsia.

[20] Algattas H, Huang JH (2014). Traumatic brain injury pathophysiology and treatments: early, intermediate, and late phases post-injury. Int J Mol Sci.

[21] Shultz SR, Wright DK, Zheng P, Stuchbery R, Liu SJ, Sashindranath M (2015). Sodium selenate reduces hyperphosphorylated tau and improves outcomes after traumatic brain injury. Brain.

[22] Mustafa AG, Alshboul OA (2013). Pathophysiology of traumatic brain injury. Neurosciences (Riyadh).

[23] Rovegno M, Soto PA, Saez JC, von Bernhardi R (2012). Biological mechanisms involved in the spread of traumatic brain damage. Med Intensiva.

[24] Gentleman SM, Leclercq PD, Moyes L, Graham DI, Smith C, Griffin WS (2004). Long-term intracerebral inflammatory response after traumatic brain injury. Forensic Sci Int.

[25] Hausmann R, Kaiser A, Lang C, Bohnert M, Betz P (1999). A quantitative immunohistochemical study on the time-dependent course of acute inflammatory cellular response to human brain injury. Int J Legal Med.

[26] Bao F, Shultz SR, Hepburn JD, Omana V, Weaver LC, Cain DP (2012). A CD11d monoclonal antibody treatment reduces tissue injury and improves neurological outcome after fluid percussion brain injury in rats. J Neurotrauma.

[27] Kumar A, Loane DJ (2012). Neuroinflammation after traumatic brain injury: opportunities for therapeutic intervention. Brain Behav Immun.

[28] Lozano D, Gonzales-Portillo GS, Acosta S, de la Pena I, Tajiri N, Kaneko Y (2015). Neuroinflammatory responses to traumatic brain injury: etiology, clinical consequences, and therapeutic opportunities. Neuropsychiatr Dis Treat.

[29] Pape HC, Marcucio R, Humphrey C, Colnot C, Knobe M, Harvey EJ (2010). Trauma-induced inflammation and fracture healing. J Orthop Trauma.

[30] Zhang H, Sun T, Liu Z, Zhang J, Wang X, Liu J (2013). Systemic inflammatory responses and lung injury following hip fracture surgery increases susceptibility to infection in aged rats. Mediators Inflamm.

[31] Cibelli M, Fidalgo AR, Terrando N, Ma D, Monaco C, Feldmann M (2010). Role of interleukin-1beta in postoperative cognitive dysfunction. Ann Neurol.

[32] Lee JS, Ryu CH, Moon NH, Kim SJ, Park SY, Suh KT (2009). Changes in serum levels of receptor activator of nuclear factor-kappaB ligand, osteoprotegerin, IL-6 and TNF-alpha in patients with a concomitant head injury and fracture. Arch Orthop Trauma Surg.

[33] Terrando N, Monaco C, Ma D, Foxwell BM, Feldmann M, Maze M (2010). Tumor necrosis factor-alpha triggers a cytokine cascade yielding postoperative cognitive decline. Proc Natl Acad Sci U S A.

[34] Wilson M, Davis DP, Coimbra R (2003). Diagnosis and monitoring of hemorrhagic shock during the initial resuscitation of multiple trauma patients: a review. J Emerg Med.

[35] Keel M, Trentz O (2005). Pathophysiology of polytrauma. Injury.

[36] Shaikh N (2009). Emergency management of fat embolism syndrome. J Emerg Trauma Shock.

[37] Pape HC, Giannoudis P, Krettek C (2002). The timing of fracture treatment in polytrauma patients: relevance of damage control orthopedic surgery. Am J Surg.

[38] Prasad G, Dhillon MS, Khullar M, Nagi ON (2003). Evaluation of oxidative stress after fractures. A preliminary study. Acta Orthop Belg.

[39] Weiss S, Zimmermann G, Pufe T, Varoga D, Henle P (2009). The systemic angiogenic response during bone healing. Arch Orthop Trauma Surg.

[40] Bolander ME (1992). Regulation of fracture repair by growth factors. Proc Soc Exp Biol Med.

[41] Xiong Y, Mahmood A, Chopp M (2013). Animal models of traumatic brain injury. Nat Rev Neurosci.

[42] Lefering R, Paffrath T, Linker R, Bouillon B, Neugebauer EA, Deutsche Gesellschaft fur Unfallchirurgie/German Society for Trauma S (2008). Head injury and outcome—what influence do concomitant injuries have?. J Trauma.

[43] Leitgeb J, Mauritz W, Brazinova A, Majdan M, Wilbacher I (2013). Impact of concomitant injuries on outcomes after traumatic brain injury. Arch Orthop Trauma Surg.

[44] Siegel JH, Gens DR, Mamantov T, Geisler FH, Goodarzi S, MacKenzie EJ (1991). Effect of associated injuries and blood volume replacement on death, rehabilitation needs, and disability in blunt traumatic brain injury. Crit Care Med.

[45] van Leeuwen N, Lingsma HF, Perel P, Lecky F, Roozenbeek B, Lu J (2012). Prognostic value of major extracranial injury in traumatic brain injury: an individual patient data meta-analysis in 39,274 patients. Neurosurgery.

[46] Leong BK, Mazlan M, Abd Rahim RB, Ganesan D (2013). Concomitant injuries and its influence on functional outcome after traumatic brain injury. Disabil Rehabil.

[47] Lingsma H, Andriessen TM, Haitsema I, Horn J, van der Naalt J, Franschman G (2013). Prognosis in moderate and severe traumatic brain injury: external validation of the IMPACT models and the role of extracranial injuries. J Trauma Acute Care Surg.

[48] Hensler T, Sauerland S, Bouillon B, Raum M, Rixen D, Helling HJ (2002). Association between injury pattern of patients with multiple injuries and circulating levels of soluble tumor necrosis factor receptors, interleukin-6 and interleukin-10, and polymorphonuclear neutrophil elastase. J Trauma.

[49] Savola O, Pyhtinen J, Leino TK, Siitonen S, Niemela O, Hillbom M (2004). Effects of head and extracranial injuries on serum protein S100B levels in trauma patients. J Trauma.

[50] Baltas I, Gerogiannis N, Sakellariou P, Matamis D, Prassas A, Fylaktakis M (1998). Outcome in severely head injured patients with and without multiple trauma. J Neurosurg Sci.

[51] Sarrafzadeh AS, Peltonen EE, Kaisers U, Kuchler I, Lanksch WR, Unterberg AW (2001). Secondary insults in severe head injury—do multiply injured patients do worse?. Crit Care Med.

[52] Stulemeijer M, van der Werf SP, Jacobs B, Biert J, van Vugt AB, Brauer JM (2006). Impact of additional extracranial injuries on outcome after mild traumatic brain injury. J Neurotrauma.

[53] Kumar RG, Diamond ML, Boles JA, Berger RP, Tisherman SA, Kochanek PM (2015). Acute CSF interleukin-6 trajectories after TBI: associations with neuroinflammation, polytrauma, and outcome. Brain Behav Immun.

[54] Hukkelhoven CW, Steyerberg EW, Rampen AJ, Farace E, Habbema JD, Marshall LF (2003). Patient age and outcome following severe traumatic brain injury: an analysis of 5600 patients. J Neurosurg.

[55] Bagiella E, Novack TA, Ansel B, Diaz-Arrastia R, Dikmen S, Hart T (2010). Measuring outcome in traumatic brain injury treatment trials: recommendations from the traumatic brain injury clinical trials network. J Head Trauma Rehabil.

[56] Shukla D, Devi BI, Agrawal A (2011). Outcome measures for traumatic brain injury. Clin Neurol Neurosurg.

[57] Stein DG (2015). Embracing failure: what the phase III progesterone studies can teach about TBI clinical trials. Brain Inj.

[58] Green SM (2011). Cheerio, laddie! Bidding farewell to the Glasgow Coma Scale. Ann Emerg Med.

[59] DeWitt DS, Jenkins LW, Prough DS (1995). Enhanced vulnerability to secondary ischemic insults after experimental traumatic brain injury. New Horiz.

[60] McHugh GS, Engel DC, Butcher I, Steyerberg EW, Lu J, Mushkudiani N (2007). Prognostic value of secondary insults in traumatic brain injury: results from the IMPACT study. J Neurotrauma.

[61] Selassie AW, Fakhry SM, Ford DW (2011). Population-based study of the risk of in-hospital death after traumatic brain injury: the role of sepsis. J Trauma.

[62] Stocchetti N, Taccone FS, Citerio G, Pepe PE, Le Roux PD, Oddo M (2015). Neuroprotection in acute brain injury: an up-to-date review. Crit Care.

[63] Gebhard F, Huber-Lang M (2008). Polytrauma—pathophysiology and management principles. Langenbecks Arch Surg.

[64] Flierl MA, Stoneback JW, Beauchamp KM, Hak DJ, Morgan SJ, Smith WR (2010). Femur shaft fracture fixation in head-injured patients: when is the right time?. J Orthop Trauma.

[65] Lenz A, Franklin GA, Cheadle WG (2007). Systemic inflammation after trauma. Injury.

[66] Weaver LC, Bao F, Dekaban GA, Hryciw T, Shultz SR, Cain DP (2015). CD11d integrin blockade reduces the systemic inflammatory response syndrome after traumatic brain injury in rats. Exp Neurol.

[67] Shlosberg D, Benifla M, Kaufer D, Friedman A (2010). Blood-brain barrier breakdown as a therapeutic target in traumatic brain injury. Nat Rev Neurol.

[68] Chodobski A, Zink BJ, Szmydynger-Chodobska J (2011). Blood-brain barrier pathophysiology in traumatic brain injury. Transl Stroke Res.

[69] Lucas SM, Rothwell NJ, Gibson RM (2006). The role of inflammation in CNS injury and disease. Br J Pharmacol.

[70] Hergenroeder GW, Moore AN, McCoy JP, Samsel L, Ward NH, Clifton GL (2010). Serum IL-6: a candidate biomarker for intracranial pressure elevation following isolated traumatic brain injury. J Neuroinflammation.

[71] Vester H, Huber-Lang MS, Kida Q, Scola A, van Griensven M, Gebhard F (2014). The immune response after fracture trauma is different in old compared to young patients. Immun Ageing.

[72] Woodcock T, Morganti-Kossmann MC (2013). The role of markers of inflammation in traumatic brain injury. Front Neurol.

[73] Agoston DV, Elsayed M (2012). Serum-based protein biomarkers in blast-induced traumatic brain injury spectrum disorder. Front Neurol.

[74] Shultz SR, Tan XL, Wright DK, Liu SJ, Semple BD, Johnston L (2014). Granulocyte-macrophage colony-stimulating factor is neuroprotective in experimental traumatic brain injury. J Neurotrauma.

[75] Jones NC, Prior MJ, Burden-Teh E, Marsden CA, Morris PG, Murphy S (2005). Antagonism of the interleukin-1 receptor following traumatic brain injury in the mouse reduces the number of nitric oxide synthase-2-positive cells and improves anatomical and functional outcomes. Eur J Neurosci.

[76] Longhi L, Perego C, Ortolano F, Aresi S, Fumagalli S, Zanier ER (2013). Tumor necrosis factor in traumatic brain injury: effects of genetic deletion of p55 or p75 receptor. J Cereb Blood Flow Metab.

[77] Maegele M, Riess P, Sauerland S, Bouillon B, Hess S, McIntosh TK (2005). Characterization of a new rat model of experimental combined neurotrauma. Shock.

[78] Maegele M, Sauerland S, Bouillon B, Schafer U, Trubel H, Riess P (2007). Differential immunoresponses following experimental traumatic brain injury, bone fracture and “two-hit”-combined neurotrauma. Inflamm Res.

[79] Probst C, Mirzayan MJ, Mommsen P, Zeckey C, Tegeder T, Geerken L (2012). Systemic inflammatory effects of traumatic brain injury, femur fracture, and shock: an experimental murine polytrauma model. Mediators Inflamm.

[80] Weckbach S, Perl M, Heiland T, Braumuller S, Stahel PF, Flierl MA (2012). A new experimental polytrauma model in rats: molecular characterization of the early inflammatory response. Mediators Inflamm.

[81] Weckbach S, Hohmann C, Braumueller S, Denk S, Klohs B, Stahel PF (2013). Inflammatory and apoptotic alterations in serum and injured tissue after experimental polytrauma in mice: distinct early response compared with single trauma or “double-hit” injury. J Trauma Acute Care Surg.

[82] Hang CH, Shi JX, Tian J, Li JS, Wu W, Yin HX (2004). Effect of systemic LPS injection on cortical NF-kappaB activity and inflammatory response following traumatic brain injury in rats. Brain Res.

[83] Utagawa A, Truettner JS, Dietrich WD, Bramlett HM (2008). Systemic inflammation exacerbates behavioral and histopathological consequences of isolated traumatic brain injury in rats. Exp Neurol.

[84] Mirzayan MJ, Probst C, Samii M, Krettek C, Gharabaghi A, Pape HC (2012). Histopathological features of the brain, liver, kidney and spleen following an innovative polytrauma model of the mouse. Exp Toxicol Pathol.

[85] Shultz SR, Sun M, Wright DK, Brady RD, Liu S, Beynon S (2015). Tibial fracture exacerbates traumatic brain injury outcomes and neuroinflammation in a novel mouse model of multitrauma. J Cereb Blood Flow Metab.

[86] Yang L, Guo Y, Wen D, Yang L, Chen Y, Zhang G (2016). Bone fracture enhances trauma brain injury. Scand J Immunol.

[87] Degos V, Maze M, Vacas S, Hirsch J, Guo Y, Shen F (2013). Bone fracture exacerbates murine ischemic cerebral injury. Anesthesiology.

[88] Han Z, Li L, Wang L, Degos V, Maze M, Su H (2014). Alpha-7 nicotinic acetylcholine receptor agonist treatment reduces neuroinflammation, oxidative stress, and brain injury in mice with ischemic stroke and bone fracture. J Neurochem.

[89] Ramlackhansingh AF, Brooks DJ, Greenwood RJ, Bose SK, Turkheimer FE, Kinnunen KM (2011). Inflammation after trauma: microglial activation and traumatic brain injury. Ann Neurol.

[90] Coughlin JM, Wang Y, Munro CA, Ma S, Yue C, Chen S (2015). Neuroinflammation and brain atrophy in former NFL players: an in vivo multimodal imaging pilot study. Neurobiol Dis.

[91] Borovikova LV, Ivanova S, Zhang M, Yang H, Botchkina GI, Watkins LR (2000). Vagus nerve stimulation attenuates the systemic inflammatory response to endotoxin. Nature.

[92] Lee DY, Cho TJ, Kim JA, Lee HR, Yoo WJ, Chung CY (2008). Mobilization of endothelial progenitor cells in fracture healing and distraction osteogenesis. Bone.

[93] Laing AJ, Dillon JP, Condon ET, Street JT, Wang JH, McGuinness AJ (2007). Mobilization of endothelial precursor cells: systemic vascular response to musculoskeletal trauma. J Orthop Res.

